# Development of a patient-specific chest computed tomography imaging phantom with realistic lung lesions using silicone casting and three-dimensional printing

**DOI:** 10.1038/s41598-023-31142-5

**Published:** 2023-03-09

**Authors:** Dayeong Hong, Sojin Moon, Joon Beom Seo, Namkug Kim

**Affiliations:** 1 Department of Radiological Science, Dongnam Health University, 50 Cheoncheon-Ro 74 Gil, Jangan-Gu, Suwon-Si, Gyeonggi-Do 16328 Republic of Korea; 2 Department of Radiology, University of Ulsan College of Medicine, Asan Medical Center, 88 Olympic-Ro 43 Gil, Songpa-Gu, Seoul, Republic of Korea; 3 Department of Radiology and Convergence Medicine, AMIST, Asan Medical Center, University of Ulsan College of Medicine, 88 Olympic-Ro 43 Gil, Songpa-Gu, Seoul, 05505 South Korea

**Keywords:** Biological techniques, Biotechnology, Anatomy, Diseases

## Abstract

The validation of the accuracy of the quantification software in computed tomography (CT) images is very challenging. Therefore, we proposed a CT imaging phantom that accurately represents patient-specific anatomical structures and randomly integrates various lesions including disease-like patterns and lesions of various shapes and sizes using silicone casting and three-dimensional (3D) printing. Six nodules of various shapes and sizes were randomly added to the patient’s modeled lungs to evaluate the accuracy of the quantification software. By using silicone materials, CT intensities suitable for the lesions and lung parenchyma were realized, and their Hounsfield unit (HU) values were evaluated on a CT scan of the phantom. As a result, based on the CT scan of the imaging phantom model, the measured HU values for the normal lung parenchyma, each nodule, fibrosis, and emphysematous lesions were within the target value. The measurement error between the stereolithography model and 3D-printing phantoms was 0.2 ± 0.18 mm. In conclusion, the use of 3D printing and silicone casting allowed the application and evaluation of the proposed CT imaging phantom for the validation of the accuracy of the quantification software in CT images, which could be applied to CT-based quantification and development of imaging biomarkers.

## Introduction

The use of quantitative computed tomography (CT) for diagnosing lung diseases is expanding each day, and it is applied to various lung diseases. In particular, research related to lung diseases has increased because of the outbreak of the coronavirus disease-2019, and most studies are conducted using CT images^1–3^.

The verification of the intensity correction and quantitative measurement of CT images is a very important topic. Although various types of CT imaging phantoms have been developed in the past, the development of CT chest imaging phantoms specific to patients and diseases has limitations. Nevertheless, many studies have focused on the fabrication of CT imaging phantoms to calibrate CT image intensity and validate the accuracy of the quantitative measurement software. In particular, patient-specific and disease-specific three-dimensional (3D) printing is possible compared with existing processing technologies, and complex designs can be prototyped quickly. 3D printing can apply various models to medical care, such as for education and surgical guide^4–13^. Many studies have also conducted phantom fabrication for imaging quantification^14,15^. Hong et al. developed a disease-specific lung imaging phantom using 3D printing^16^. Shin et al. used 3D printing to develop a reproducible, deformable lung phantom with 3D-printed airways^17^. Hazelaar et al. produced a phantom to evaluate the X-ray-based image quality and position verification technique for radiotherapy that is very similar to the actual patient^18^. Kairn et al. developed a phantom using a tissue-compatible material with a single 3D printer^19^. Filippou et al. created an advanced phantom using various medical images^20^. Although various types of patient- and disease-specific phantoms have been developed, the development of a standard imaging phantom that can determine the measurement accuracy of each imaging phantom remains a challenge.

Chest CT intensities reflect various anatomical objects including the airways, lung parenchyma, fats, soft tissues, and bones. In addition, lesions with different patterns can develop in the lung parenchyma. However, quantitative CT depends on factors such as imaging protocols, reconstruction parameters, patients’ motion, and CT artifacts, except CT intensities. Therefore, the reliability of quantitative chest CT should be evaluated by using patient- and disease-specific imaging phantom. This study aimed to fabricate a chest CT imaging phantom that reflects the CT intensity of various lung lesions using 3D-printing technology and silicone casting and to evaluate its quantification accuracy.

## Methods

This retrospective study was conducted in accordance with the principles of the Declaration of Helsinki and current scientific guidelines. The study protocol was approved by the Institutional Review Board of Asan Medical Center, South Korea. The requirement of informed consent from images was waived by the Institutional Review Board of Asan Medical Center (AMC). All methods were performed in accordance with the relevant guidelines and regulations.

**Figure 1:**
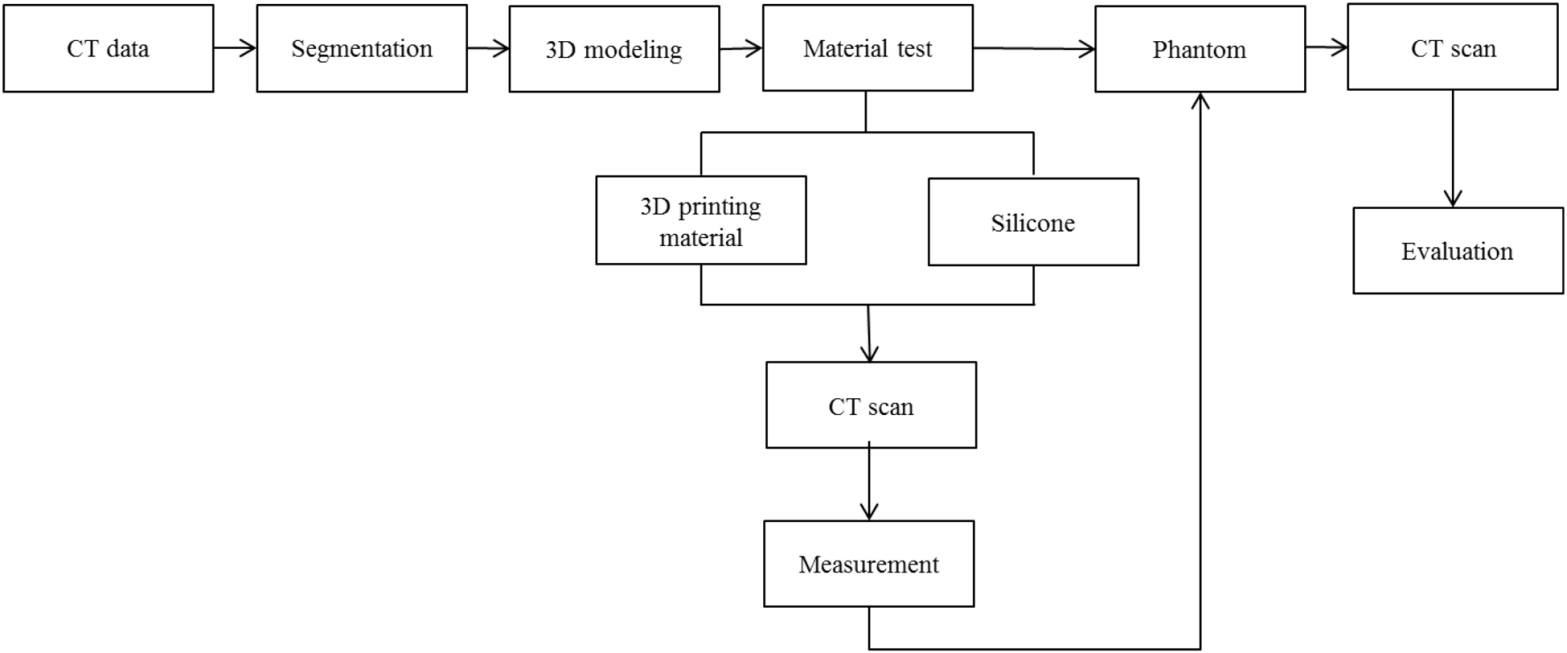
Overall workflow for fabricating a chest imaging phantom using three-dimensional printing and silicone casting.

To produce a CT imaging phantom using 3D printing, studies on Hounsfield unit (HU) values have used various silicone materials as 3D-printing materials. Materials research was conducted based on the shapes shown in HU and CT images of various materials. Various lesions were then extracted from the chest CT scans of the patients, and phantoms were manufactured using appropriate materials. HU evaluation was based on the well-known HU value of each anatomical structure of the human body^21,22^. The measurement errors of the size between the reference and measured CT values of the inner diameter of the right ventricle, solid nodule, part of the lung vessel, and part of the airway were evaluated. The measured sizes were then analyzed using the Bland–Altman method. The overall workflow is shown in Fig. 1.

### Medical image acquisition

An anonymous patient, various silicone samples for fabricating phantoms, and 3D-printed chest phantoms were scanned using a dual-source CT (SOMATOM Definition Flash, Siemens Healthcare) with a standard protocol of 120 kVp and 1.0 mm slice thickness. These scan data were also reconstructed to 0.6 mm in the axial section using software (Syngo CT 2012B).

### Phantom design

**Figure 2:**
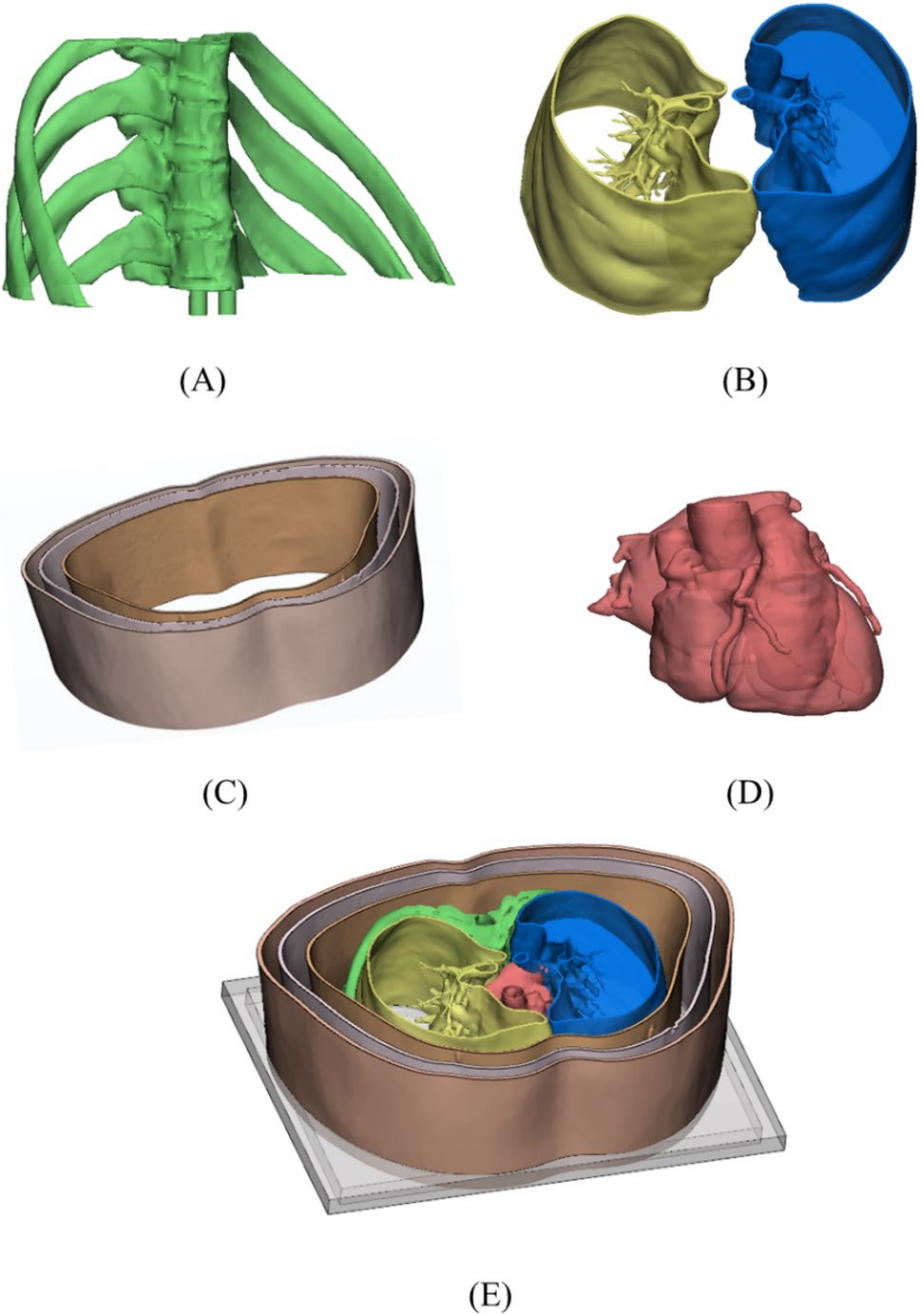
3D modeling of chest CT image phantom based in CT images of a patient. (**A**) Spine and rib, (**B**) left and right lungs, (**C**) molder of the skin, fat, and muscle, (**D**) heart, and (**E**) composition of (**A**–**D**). CT, computed tomography; 3D, three-dimensional.

The developed phantom reflects the human anatomy based on the chest CT images of a patient. The lung lobes, spine, ribs, heart, fat, and skin were designed (Fig. 2). These anatomical structures were segmented using the medical image segmentation program Mimics software (Materialise Inc., Louvain, Belgium). For the design of the phantom model, a part of the chest CT section was modeled with 3-matic software (Materialise Inc.). Normal lung parenchyma and emphysema in the right lobe and solid nodule and fibrosis lesion in the left lobe were placed randomly. In addition, a thoracic cross-sectional model that included the heart, aorta, vertebrae, and ribs surrounding the lungs was made. Various anatomical structures were designed to be assembled on the lower plate in a negatively embossed manner. The skin, fats, and muscles were then made into separate layers to reflect the characteristics of each anatomy.

### 3D-printing materials

The phantom molder was made using 3D-printing materials. The molder should be strong enough to avoid leakage of silicone, withstand the expansion force of silicone, and produce the chest CT axial phase of an actual adult. Therefore, robust and economical acrylonitrile butadiene styrene (ABS) material of fused deposition modeling (FDM) was selected and printed by Stratasys Fortus 900MC^23^. In addition, the heart model reflected the shape of a real heart using flexible thermoplastic polyurethane (TPU) material of FDM (Ultimaker S5, Ultimaker) regardless of HU. The spine and rib were printed using polylactic acid materials of hydrophilic FDM (Ultimaker S5, Ultimaker) for HU implementation. Then, it was immersed in the contrast medium (Ultravist 370 mg I/mL; Bayer Healthcare, Berlin, Germany) for 48 h so that the printed material could absorb the contrast medium.

### Silicone materials

First, to implement the pattern of the alveoli of the lung parenchyma in detail, CT was performed using some silicone materials to confirm its HU value. The silicone material was obtained from Smooth-On Co. of the FlexFoam-iT! series (Table 1). These silicone materials are expandable and durable. These can have an expansion rate from as high as 15 times to as low as 2 times. Therefore, the silicone to be used for the phantom was selected based on the CT intensity and pattern of each silicone. CT intensity was based on the HU for the human body^21,22^, and the silicone pattern was selected by referring to the basic pattern corresponding to each lung lesion^24,25^. Therefore, to induce emphysema, the FlexFoam-iT! V was used in the lower right lobe of the lung, and to simulate normal lung parenchyma, FlexFoam-iT! 17 was used. In addition, to induce pulmonary fibrosis, FlexFoam-iT! 23FR was used, and the lung parenchyma containing solid nodule was tested with FlexFoam-iT! X.

**Table 1:** Summary of the silicone materials used for the chest imaging phantom.

Material	Ratio (main:hardner)	Pot life (23 °C)	Hardening time (23 °C)	Forming magnification
FlexFoam-iT! X	1:1	60 (sec)	120 (min)	6 (times)
FlexFoam-iT! V	1:1	60 (sec)	120 (min)	11 (times)
FlexFoam-iT! 17	2:1	60 (sec)	120 (min)	3.5 (times)
FlexFoam-iT! 23	8.5:10	90 (sec)	120 (min)	2 (times)
Ecoflex 0020	1:1	1800 (sec)	240 (min)	−
Dragon skin pro fx	1:1	720 (sec)	40 (min)	−

Furthermore, to realize the fat and muscle surrounding the chest, gel wax and Ecoflex0020 silicone were used. Ecoflex 0020 silicone was used after mixing the main agent and curing agent in a 1:1 ratio, and air bubbles were removed using a deaerator. In addition, silicone of Dragon Skin FX Pro was used to model the skin.

### Statistical evaluation

**Figure 3:**
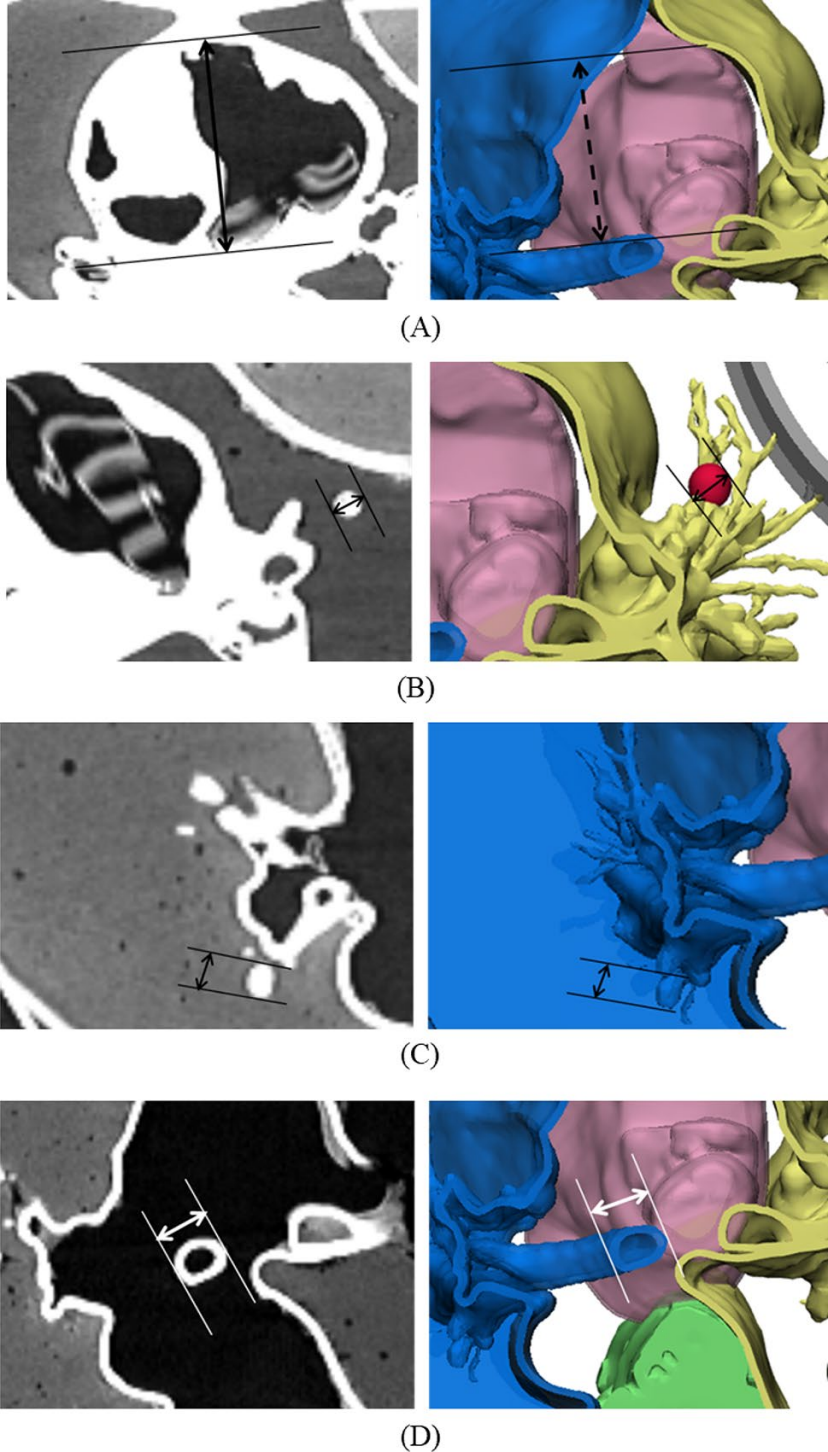
3D-modeled Standard Tessellation Language (STL) and CT of 3D-printed phantom with landmarks specified for evaluating measurement error. (**A**) Inner diameter of the right ventricle, (**B**) solid nodule, (**C**) part of a lung vessel, and (**D**) outer diameter of the airway. CT, computed tomography; 3D, three-dimensional.

The CT value range was evaluated to compare the accuracy of the designed Standard Tessellation Language (STL) model and the measurements in the CT image of the 3D-printed phantom. All measurements were repeated five times each by one observer. For HU evaluation, the HU values of the normal lung parenchyma, lung diseases (fibrosis, solid nodule, and emphysema), and chest structures (muscle, fat, skin, and bone) were compared. To measure the shape accuracy, one part was selected from each anatomical region, and the length was measured using RadiAnt DICOM viewer (Medixant Inc., Poznan, Poland). STL images for 3D printing and CT images of the 3D-printed phantoms were measured (Fig. 3).

## Results

### Baseline HU evaluation with different silicone materials

This study used a two-component silicone material that foams when the first and second agents are mixed. The degree of foaming varied with the type used, and as the silicone has different porosities, it was suitable for creating various patterns of lungs containing air. To realize the chest CT imaging phantom, various silicone patterns and HUs were identified. Silicone materials with various patterns and CT values were used to develop thoracic phantoms with various internal HU (Table 2).

**Table 2:** Lung structures matched with the HU of the FlexFoam-iT.

Material	HU	Lung structure
FlexFoam-iT! X	− 807.42 ± 5.71	Lung parenchyma with high HU
FlexFoam-iT! V	− 885.82 ± 8.52	Emphysema
FlexFoam-iT! 17	− 651.01 ± 15.97	Lung parenchyma with low HU
FlexFoam-iT! 23	− 544.97 ± 13.63	Fibrosis

HU, Hounsfield unit.

### Chest imaging phantom for CT

**Figure 4:**
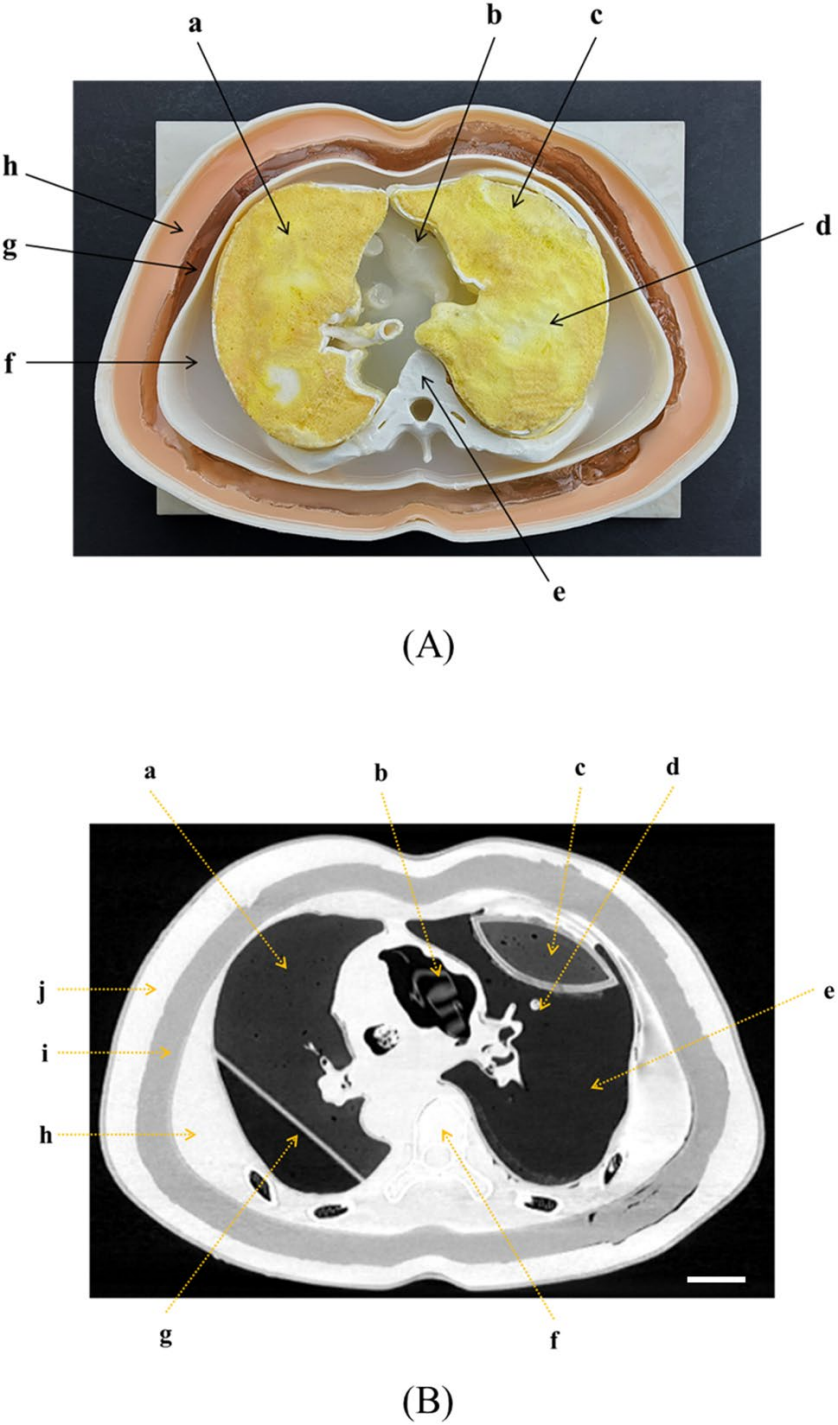
3D printing image phantom and CT image. (**A**) Fabrication of the chest phantom using 3D-printing technology (a, emphysema; b, heart; c, fibrosis; d, normal lung parenchyma; e, spine and rib; f, muscle; g, fat; and h, skin), and (**B**) CT image of the phantom (a, emphysema; b, heart; c, fibrosis; d, solid nodule; e, normal lung parenchyma; f, spine and rib; g, fissure; h, muscle; i, fat; and j, skin). CT, computed tomography; 3D, three-dimensional.

Based on the aforementioned 3D printing and silicone casting, an axial section of the chest was taken from a CT image of a patient to produce a disease-specific chest imaging phantom. The lung lobes, heart, airways, muscle layers, fat layers, skin, ribs, and spine were modeled (Fig. 4). In addition, various lung lesions were randomly constructed. The chest imaging phantom was made using various 3D-printing materials, patterns, and silicone materials. The HU values of the lung parenchyma, lung lesions, muscles, and fat layers as well as the morphology of the spine and ribs were realized in the CT image of the phantom. In addition, a shape similar to the axial phase of the human chest CT was modeled.

**Figure 5:**
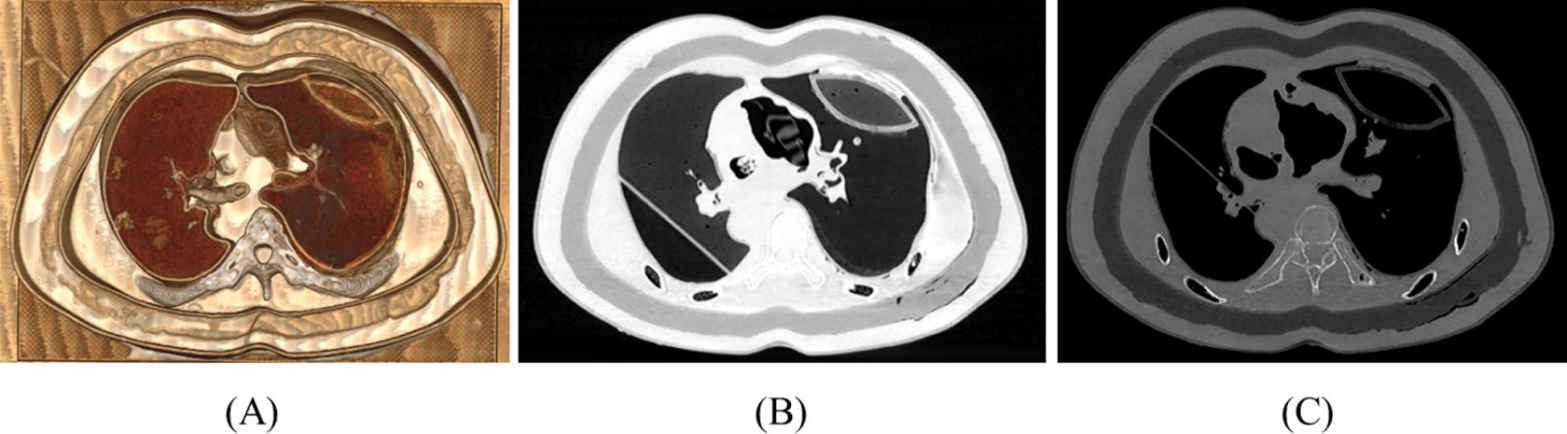
Various CT image settings of the phantoms. (**A**) CT volume-rendering image of the phantom, (**B**) CT image of the phantom with lung window setting, and (**C**) CT image of the phantom with bone window setting. CT, computed tomography.

The human lung, which mainly contained air, had HU of − 600 to − 800 under normal conditions, − 850 to − 950 in the case of emphysema, − 500 to − 700 in pulmonary fibrosis, and − 100 in solid nodules, and similar HU is implemented using various silicone materials. In an actual human body, the muscle and fat surrounding the lungs have 10–150 HU and − 100, respectively, and the phantom also had similar values. Moreover, the phantom reflects the visual merit by reflecting the hue and color of the external skin similar to the actual human body (Table 3 and Fig. 5).

**Table 3:** Comparison of the HU values between the CT image and 3D-printed phantom^21,22^.

		Standard value	Phantom value
Normal lung parenchyma		− 600 to − 800	− 777.32 ± 24.84
Lung disease	Fibrosis	− 500 to − 700	− 682.61 ± 22.92
	Solid nodule	~ − 200	− 120.05 ± 41.86
	Emphysema	− 850 ~	− 908.55 ± 18.32
Chest structure	Muscle	10 to 150	111.33 ± 23.22
	Fat	− 50 to − 100	− 159.60 ± 20.98
	Skin	− 200 to + 100	164.75 ± 28.92
	Bone	> 1000	199.80 ± 24.14

**Figure 6:**
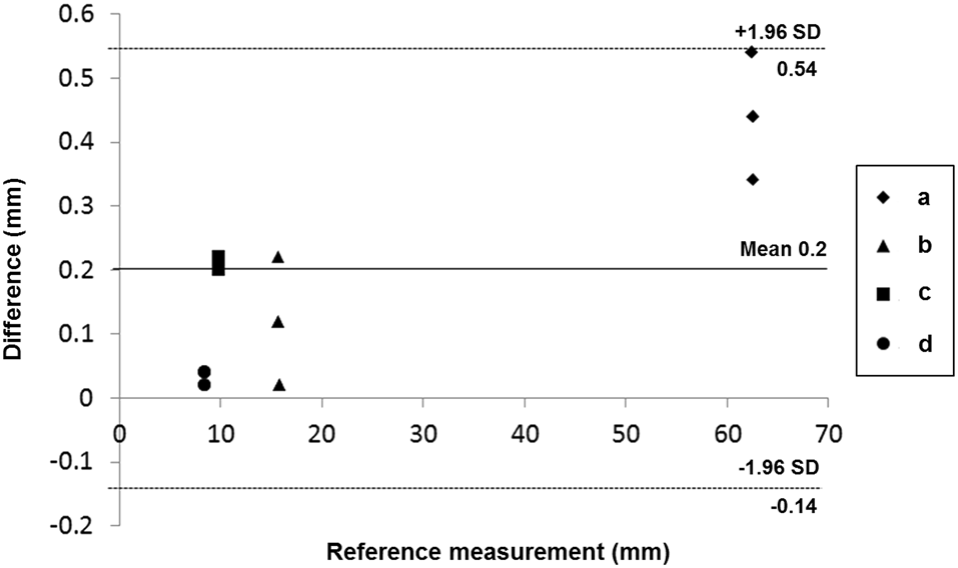
Bland–Altman analysis used to evaluate differences between the 3D-modeled STL (standard) and the CT from the printed phantom. (**a**) Inner diameter of the right ventricle, (**b**) solid nodule, (**c**) part of a lung vessel, and (**d**) part of the airway. CT, computed tomography; 3D, three-dimensional.

The corresponding landmarks of the anatomical structures between the 3D-modeled STL and the CT image of printed phantoms were compared based on the measurements obtained and were evaluated using a Bland–Altman plot. The mean ± standard deviation of the differences was 0.20 ± 0.19 mm (limits of agreement, from − 0.1 to 0.5 mm) (Fig. 6).

## Discussion

The existing commercialized CT imaging phantom is mainly used for the calibration of CT intensity, equipment maintenance, repair, and regular evaluation. Conventional phantoms also have some limitations, as they are not customized to each patient and disease, are expensive, and not realistic^26,27^. Therefore, our study focused on the fabrication of a patient- and disease-specific imaging phantom using 3D-printing technology that can overcome the limitations of conventional phantoms. The use of 3D-printing technology with various materials can simulate the CT intensity of various lesions, and the size, shape, and number of lesions can be realized. Therefore, 3D-printing technology makes it possible to easily manufacture patient-specific and disease-specific imaging phantoms. For this reason, Sindi et al. used silicone and peanut oils to fabricate a patient-specific 3D-printed breast phantom for magnetic resonance imaging^28^. However, the photopolymer resin as 3D-printing material may be deformed. Buytaert et al. used 3D printing to fabricate a patient-specific phantom to simulate the frame for coronary angiography^29^. However, their phantom cannot reflect depth information as a medical image phantom because only a limited cross-section of the image was shaped. Legnani et al. developed a vascular 3D phantom for stereotactic radiosurgery of arteriovenous malformations^30^. This study suggests that the proposed method has the potential for producing patient-specific models for neurovascular radiosurgery applications and medical research.

In the present study, CT chest phantoms were developed to reflect various lung lesions with actual CT intensities and validate the accuracy of the quantitative measurements of the software. A molder for the chest phantom was made using ABS with 0–200 HU, and the spine and ribs were printed using hydrophilic polylactic acid (PLA), which is expected to absorb the contrast agent and has CT intensity similar to bones. In addition, the heart anatomy was printed using flexible TPU material so that it could be fixed into the chest phantom. By using silicone materials with foaming characteristics, various patterns of the normal parenchyma and lesions with actual CT intensity were made. The HU values of the normal lung parenchyma and emphysema, solid nodule, and fibrosis ranged from − 800 to − 600, -850 to − 950, 100 to -200, and − 500 to − 700, respectively.

The strength of this study is attributed to the modeling of realistic lung lesions. The use of 3D-printing technology to create an imaging phantom helped overcome the limitations of existing commercialized phantoms. Many chest phantom studies have been conducted. Mei et al. demonstrated the feasibility of 3D-printed patient-based lung phantoms with accurate organ geometry, image texture, and attenuation profiles^31^. This study succeeded in realizing a part of a normal lung with CT intensity implemented using the pixel 3D-printing method. In addition, Hernandez-Giron et al. fabricated a 3D-printed anthropomorphic lung phantom for image quality assessment in CT, but its shape was very different from the patient anatomy^32^. In this study, the phantom evaluated the dose characteristics of the CT image, but the shape of the phantom was different from that of an actual human. Zhang et al. fabricated a personalized anthropomorphic phantom using 3D printing and tissue-compatible materials^33^. Craft and Howell prepared and fabricated a full-scale, sagittal-sliced, 3D-printed, patient-specific radiotherapy phantom^34^. However, these studies have some limitations in view of the realistic texture and shape of various lung lesions with actual CT intensity.

In this study, CT HU values of various lung lesions were represented using silicone materials. The FDM 3D printer is the most economical and accessible printing method, which could be one of its advantages for actual clinical applications. Developing a phantom with similar CT intensity with an exact anatomical shape that represents the human body enables quantitative evaluation of CT software in realistic situations. It is also useful for educational purposes. With the CT image of the phantom that presents various lesions, the training efficiency of image reading for radiologists could be increased. In addition, a patient-specific model can help clinicians smoothly educate and communicate with patients about their diseases.

This study has several limitations. First, the CT intensity was not representative because the contrast medium was not absorbed well. In the future, the desired value will be reflected by mixing the appropriate amount of metallic FDM filament. Second, the shape of the heart was not accurate in the axial section of the chest CT image. To present the exact shape of the heart, additional research is needed to produce a similar image. Third, silicone was used to model various lung lesions inside the lung, but the 3D-printed molder used for the location of the silicone cannot be manually removed. It was difficult to remove the molder because of the viscosity of the silicone used, which can be overcome by using silicone-releasing agents. Fourth, silicone was used by mixing and foaming the first and second agents. The ratio and pot life of the first and second agents may vary depending on individual mixing; thus, the porosity may change. In the future, this problem may be overcome through automation and mechanization of the silicone-mixing process. Fifth, the length measured between the 3D model and 3D-printed phantom CT image may differ depending on the thresholding value of the CT image^35^. Therefore, since the value can change depending on the boundary between the inner and outer surfaces of the same structure, reproducibility can be maintained by measuring the same image setting value of the CT image. In conclusion, using 3D-printing technology and silicone casting, we created a patient- and disease-specific chest imaging phantom that presents the CT intensity of lung lesions and shape of the actual human chest. In addition, various porous structures could be created using silicone castings to model lung lesions realistically. Unlike previous studies, a more realistic phantom was fabricated by reflecting various human structures on an axial section of the chest CT, which could be used for the evaluation of quantification software and CT intensity calibration.

## Data Availability

The datasets used and/or analysed during the current study available from the corresponding author on reasonable request.
